# Prolonged Fever; a Case Report of Medical Malpractice

**DOI:** 10.22037/aaem.v9i1.1217

**Published:** 2021-07-03

**Authors:** Fares Najari, Nasser Malekpour-Alamdari, Ideh Baradaran Kial, Dorsa Najari, Sahar Mirzaei

**Affiliations:** 1 Department of Forensic Medicine, School of Medicine, Shahid Beheshti University of Medical Sciences, Tehran, Iran.; 2 Department of General Surgery, Shahid Beheshti University of Medical Sciences, Tehran, Iran.; 3 Karaj Forensic Medicine Organization, Karaj, Iran.; 4 School of Medicine, Shahid Beheshti University of Medical Sciences, Tehran, Iran.; 5 School of Nursing and Midwifery, Shahid Beheshti University of Medical Sciences, Tehran, Iran.

**Keywords:** Catheters, catheterization, central venous, malpractice, renal dialysis, foreign bodies, fever

## Abstract

Any surgical or preoperative treatment and diagnostic procedure may be associated with complications and risks. Therefore, introduction of complicated cases plays an important role in educating those involved in the diagnosis of patients. Generally, if a physician or a nurse is informed that an item is inadvertently left behind in a patient's body during surgery, he/she is obliged to take action by notifying the healthcare system authorities and informing the patient as soon as possible; otherwise, he/she has committed a disciplinary violation. Here we present a 27-year-old female patient with a history of renal failure with prolonged fever following a retained Shaldon catheter in a patient’s chest.

## 1. Introduction:

Many critically ill patients require invasive medical procedures annually; one of which is intravenous catheterization, with a complication frequency of 15%, as reported in previous studies (1). Central venous catheterization is an important part of care in the intensive care units (ICUs), emergency wards, and operating rooms (2). It is widely used to administer antibiotics and chemotherapy drugs, provide parenteral nutrition and high-flow access for hemodialysis or plasmapheresis, and monitor the central venous pressure. Central venous catheterization is also associated with emergency complications, such as bleeding from the catheter site, pneumothorax, hemothorax, ruptured artery, catheter dislocation and displacement, and embolism (3). However, a limited number of cases of retained iatrogenic catheters as foreign bodies have been reported so far (4). Also, few cases of retained hemodialysis catheters have been reported. Here we present a 27-year-old female patient with a history of renal failure with prolonged fever following a retained Shaldon catheter in a patient’s chest. 

## 2. Case Presentation:

A 27-year-old female patient (height: 170 cm; weight: 60 kg) was referred to our internal medicine clinic with complaints of fever, weakness, lethargy, and anorexia. She had developed fever ten months before attending the clinic, mostly in the evening or at night. She reported gradual, but progressive weakness, lethargy, and anorexia, with a 7-kg weight loss in the past month. She had undergone hemodialysis in another center over the past ten months (three times a week) due to advanced renal failure. She had also been diagnosed with hyperlipidemia and hypertension about ten years ago, which were controlled with Losartan (50 mg twice a day) and Lasix (40 mg daily). She also had a history of suspected pyelonephritis before the age of five, but could not remember the treatment details. 

The hemodialysis procedure had been initiated with the implantation of a Shaldon catheter into the right jugular vein; however, due to the catheter dysfunction, a Permacath was inserted at the same site, as reported by the patient. She had been hospitalized three times due to acute infection during the ten months of hemodialysis at the same center. She was treated with antibiotics and was discharged with good general health despite experiencing the side effects of medications. In the fourth hospitalization period, she also developed asthma after antibiotic therapy and was admitted to the internal department of another center for further examinations due to the lack of recovery and prolonged fever. 

The patient appeared ill, emaciated, and impatient in the examination, and her vital signs were as follows: blood pressure (BP): 130/85 mmHg; pulse rate (PR): 94/min; respiratory rate (RR): 24/min; T: 37.8˚C; oxygen saturation (O_2_Sat): 93%; and blood sugar (BS): 99 mg/dL. No abnormalities were found in the head, face, or neck examination (except for an old scar of Permacath placement). A 2/6 murmur was heard at the right sternal border. The lungs and the internal organs were normal in abdominal examination. The results of the laboratory tests showed hypochromic microcytic anemia, high calcitonin, leukocytosis with high neutrophil count, and high erythrocyte sedimentation rate (ESR) (table 1). Thyroid function test and urine analysis were normal. The result of triple blood and urine culture was negative. 

**Figure1:**
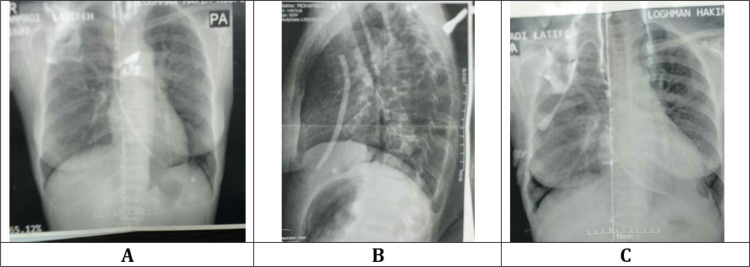
Posterior-anterior (A), Lateral (B), and anterior-posterior (C) views of patient's chest x-ray

The patient was re-examined with a possible diagnosis of subacute endocarditis. In further examinations, chest and lateral chest radiographs upon admission showed a Permacath in the posterior vena cava, with the tip positioned in the right atrium and a catheter placed parallel to the right atrium and the right ventricle with an unknown origin, without proximal connection to the implanted Permcath. Further diagnostic evaluations showed that the distal part of the Shaldon catheter implanted for hemodialysis about 10 months ago remained in chest as a foreign body (figure 1). No additional findings were reported in endoscopy of the upper gastrointestinal tract and bronchoscopy. Echocardiography also confirmed the presence of two catheters in the heart excavations. After consultation with cardiothoracic surgery specialists, the patient underwent thoracotomy, and the foreign body was removed from the right atrium. The laboratory pathology report confirmed the presence of a foreign body. All complaints and symptoms disappeared, and the patient recovered after one week. In the three-month follow-up, her general health status was good, and there was no problem, except for the end-stage renal disease (ESRD) outcome (hemodialysis three times a week).

**Table 1:** Laboratory findings of the presented case

**Test**	**Value**	**Test**	**Value**
WBC (per microliter)	13800 (71% PMN)	BUN/Cr	70/6
Hemoglobin (g/dL)	11.5	FBS (mg/dl)	146
Hematocrit (%)	28.8	Na (mEq/L)	146
Platelet (per microliter)	110000	K (mEq/L)	5.3
ESR (mm/hour)	78	Ca (mEq/L)	8
CRP	+++	P (mEq/L)	5.5
PT (ms)	14	Precalcitonin (Unit)	27
PTT (ms)	41	Total bilirubin (mg/dl)	1.8
ALT (IU/L)	25	Direct bilirubin (mg/dl)	0.2
AST (IU/L)	31	Alkaline phosphatase (IU/L)	98

WBC: white blood cell; PMN: polymorphonuclear; ESR: erythrocyte sedimentation rate; CRP: c-reactive protein; PT: prothrombin time; PTT: partial thromboplastin time; ALT: alanine aminotransferase; AST: aspartate aminotransferase; BUN: blood urea nitrogen; FBS: fasting blood sugar.

## 3. Discussion:

In this case, given the history of hemodialysis due to chronic renal failure, multiple hospitalizations, a systolic murmur in the cardiac examination, and laboratory test results (hypochromic microcytic anemia, high calcitonin, leukocytosis with a high neutrophil count, and ESR), the patient was initially diagnosed with prolonged fever due to subacute endocarditis and underwent more accurate procedures. Contrary to the opinion of the treatment staff, prolonged fever was due to a retained foreign body in the chest. As mentioned earlier, limited cases of retained catheters as foreign bodies have been reported. In this regard, Schechter et al. (2013) performed a review study of retained intravascular devices since 2000. They reviewed 115 case reports and 19 case series, comprising 574 cases of retained intravascular devices as foreign bodies, only 5.6% of which were symptomatic (4).

Moreover, Pokharel et al. (2015) performed a systematic analysis of case reports on retained central line guidewires (2). Besides, Schammer et al. (2001) reported four cases, including a 68-year-old male patient with septic shock and multiple organ failure after a rectal surgery, who underwent left femoral vein cannulation for hemofiltration by a fifth-year resident during the night shift; the routine chest radiography revealed a retained guidewire (5). In 2018, Najari et al. reported the case of a patient who underwent femoral central venous catheterization due to the lack of a suitable peripheral vein. The patient was readmitted several days later due to pain and inflammation of the lower limb, as well as hematoma on the knee. Doppler ultrasound of the lower extremity showed chronic deep vein thrombosis and several infectious areas without an echo. Radiography and MRI showed a retained guidewire in the right femoral vein (6).

Additionally, Pei-Jun Li (2016) reported a case similar to the present case. A 61-year-old female patient with uremia had undergone intermittent hemodialysis for two years. A tunneled-cuffed catheter (TCC) was implanted in the right internal jugular vein for long-term access to the central vein due to two failures in arteriovenous hemofiltration. The catheter was replaced three times due to thrombosis and infection at the catheter site. Due to dysfunction, two months after the third TCC implantation, large catheter-related thrombosis was observed on the ultrasound. During the fourth TCC replacement, the catheter was suddenly broken at the site of attachment to the skin near the right internal jugular site. In the initial attempt, the clinician tried to remove the broken piece, but failed. The emergency X-ray fluoroscopy showed two free fragments of broken TCC in the right atrium and the inferior vena cava (7).

According to a study by Schechter, most reported cases are asymptomatic, and 37% were identified accidentally during imaging for other reasons (4). There were rare cases that were symptomatic, like the present study. For example, the case reported by Hong Loan Nguyen et al. (2020) was a 39-year-old male patient admitted to a hospital in the UK due to one week of lethargy, encephalopathy, and septic shock. The chest and abdominal radiography revealed the presence of a foreign body, and the chest CT scan showed multiple bilateral, peripheral, and nodular opacities following methicillin-sensitive *Staphylococcus* *aureus* (MSSA) infection. The foreign body was observed in the sub-segmental branch of the right upper pulmonary artery. Abdominal and femoral interventions showed a left guidewire, extending from the inferior vena cava to the left common femoral vein. Further investigations revealed that the patient had been admitted to the ICU 16 years ago due to an accident and underwent central venous catheter placement in the inferior vena cava (8).

In the case presented in our study, the catheter was removed through open thoracotomy. Gabelman et al., in a review study of percutaneous retrieval and removal of the lost or misplaced intravenous instruments, reported 45 cases, including 12 retained stents, 14 retained catheters, 11 retained embolization coils, four retained left guidewires, and three retained vena cava filters (9). In our case, after discussions with experts invited to our medical team, due to the patient's complaint of the anesthesiologist’s malpractice (the person in charge of removing the Shaldon catheter), the anesthesiologist was sentenced to pay 5% of the fine. 

To prevent retained catheters, physicians and their assistant nurses should be careful about the used instruments in all medical procedures and check the items while removing them (6). It seems that use of radiography before and after catheter placement, as well as a checklist during the procedure, may help us rapidly identify and prevent similar cases.

## 4. Conclusion:

When removing vascular catheters, it is necessary to ensure the complete removal of all appendages and conduct at least one simple X-ray of the patient's chest or any other part of the patient's body involved to prevent adverse and unanticipated events and avoid the possible physical and mental consequences.

## 5. Declarations:

### 5.1 Acknowledgements

We would like to express our special thanks to the forensic center of Tehran, Iran.

### 5.2 Author’s contribution

All authors met the four criteria for authorship contribution based on the recommendations of the international committee of medical journal editors.

### 5.3 Conflict of interest

The authors declared no potential conflict of interest with respect to the authorship and/or publication of this article.

### 5.4 Funding

None.

### 5.5 Ethical considerations

 Informed consent to use the medical information was obtained from the patient at the time of discharge from the hospital.
